# Assessment of Glomerular Filtration Rate Based on Alterations of Serum Brain-Derived Neurotrophic Factor in Type 2 Diabetic Subjects Treated with Amlodipine/Benazepril or Valsartan/Hydrochlorothiazide

**DOI:** 10.1155/2015/780743

**Published:** 2015-03-30

**Authors:** I-Te Lee, Wayne Huey-Herng Sheu, Yi-Jen Hung, Jung-Fu Chen, Chih-Yuan Wang, Wen-Jane Lee

**Affiliations:** ^1^Division of Endocrinology and Metabolism, Department of Internal Medicine, Taichung Veterans General Hospital, Taichung 40705, Taiwan; ^2^School of Medicine, Chung Shan Medical University, Taichung 40201, Taiwan; ^3^School of Medicine, National Yang-Ming University, Taipei 11221, Taiwan; ^4^Division of Endocrinology and Metabolism, Department of Internal Medicine, Tri-Service General Hospital, National Defense Medical Center, Taipei 11490, Taiwan; ^5^Division of Endocrinology and Metabolism, Department of Internal Medicine, Kaohsiung Chang Gung Memorial Hospital, Kaohsiung 83301, Taiwan; ^6^Division of Endocrinology and Metabolism, Department of Internal Medicine, National Taiwan University Hospital, Taipei 10617, Taiwan; ^7^Department of Medical Research, Taichung Veterans General Hospital, Taichung 40705, Taiwan

## Abstract

*Background*. Brain-derived neurotrophic factor (BDNF) is associated with sympathetic activation. However, the effects of BDNF on diabetic nephropathy are unknown. The aim of this study was to assess the estimated glomerular filtration rates (eGFRs) and changes in serum BDNF levels in type 2 diabetic subjects treated with antihypertensive medications. *Methods*. In this randomized, double-blind clinical trial, type 2 diabetic subjects with hypertension were assigned to either the benazepril/amlodipine or valsartan/hydrochlorothiazide treatment groups for a 16-week period. The post hoc analyses were based on increased or decreased serum BDNF levels. *Results*. Of the 153 enrolled subjects, the changes in eGFR were significantly and inversely correlated with those in BDNF in the 76 subjects treated with valsartan/hydrochlorothiazide (*r* = −0.264, *P* = 0.021) but not in the 77 subjects treated with benazepril/amlodipine (*r* = −0.025, *P* = 0.862). The 45 subjects with increased BDNF following valsartan/hydrochlorothiazide treatment exhibited a significantly reduced eGFR (−8.8 ± 14.9 mL/min/1.73 m^2^; *P* < 0.001). Multivariate regression analysis revealed that increased serum BDNF represents an independent factor for reduced eGFR (95% confidence interval between −0.887 and −0.076, *P* = 0.020). *Conclusions*. Increased serum BDNF is associated with reduced eGFR in type 2 diabetic subjects treated with valsartan/hydrochlorothiazide but not with amlodipine/benazepril.

## 1. Introduction

Type 2 diabetes mellitus is a complex metabolic disorder that is associated with chronic renal complications [1]. The prevalence of diabetic nephropathy continues to increase due to expanding diabetic populations [2, 3]. Blood-pressure reduction represents an effective approach for delaying the progression of nephropathy in type 2 diabetes with hypertension [4]. However, type 2 diabetic subjects generally need to take several drugs to attain their blood-pressure goals [5].

Although fixed-dose combinations of antihypertensive medications have been recommended for efficacy and compliance, the renal protective effects might depend on the specific types of antihypertensive drugs [6, 7]. According to the Canadian Hypertension Education Program recommendations, calcium channel blockers provide more significant renal benefits than diuretics when combined with angiotensin-converting enzyme (ACE) inhibitors or angiotensin II receptor blockers (ARBs) in type 2 diabetes [7, 8]. In the Amtrel and co-Diovan in type 2 diabetes mellitus hypertension patients with microalbuminuria (ADDM) study, the amlodipine/benazepril combination more significantly maintained renal function than the valsartan/hydrochlorothiazide combination, and the benefit was independent of the blood pressure [9].

Brain-derived neurotrophic factor (BDNF) is important in neural growth and survival [10–12]. The overexpression of BDNF enhances the synaptic innervation of sympathetic neurons [13]. It has also been reported that administration of BDNF could increase the lumbar sympathetic nerve activity [14]. Renal sympathetic activation might result in reduced renal perfusion via arterial contraction [15, 16]. However, the effects elicited by circulating BDNF on renal function remain unclear. Therefore, in this study, we assessed the estimated glomerular filtration rate (eGFR) based on alterations in the serum BDNF levels in type 2 diabetic subjects treated with either amlodipine/benazepril or valsartan/hydrochlorothiazide for a 16-week period.

## 2. Subjects and Methods

### 2.1. Patients

The design of this ADDM study was detailed previously [9]. Briefly, this randomized, double-blind clinical trial was conducted at multiple centers in Taiwan. The candidates were type 2 diabetic subjects, aged 20 to 80 years, with hypertension, urinary albumin excretion (UAE, 30-to-299 mg/g) detected within one year prior to the screening period, and serum creatinine levels lower than 266 for male and 248 *μ*mol/L for female subjects, respectively, during the screening period. After screening, the enrolled subjects underwent a run-in period with a placebo treatment after discontinuing all antihypertensive drugs for a two-week period. Afterwards, the subjects were randomized to either the Amtrel (10 mg of benazepril hydrochloride/5 mg of amlodipine) or Co-Diovan (80 mg of valsartan/12.5 mg of hydrochlorothiazide) treatment group. The study was approved by the Joint Institutional Review Board of Taiwan, and written informed consent was obtained from all of the participants (clinical trial registration number: NCT01375322, ClinicalTrials.gov).

### 2.2. Biochemical Measurements

The serum concentrations of creatinine, triglyceride, total cholesterol, high-density lipoprotein (HDL), low-density lipoprotein (LDL), glucose, and BDNF were measured using blood samples collected from the subjects after an overnight fast. The glycated hemoglobin (HbA1c) levels were determined using high-performance liquid chromatography (HPLC, NGSP certified). Human BDNF was centrally measured using a commercially available immunoassay kit (R&D Systems, Minneapolis, MN, USA). The mean intra- and interassay CVs for the quantitative determination of BDNF were 4.1 and 9.0%, respectively, with a sensitivity of 0.02 ng/mL. The estimated glomerular filtration rate (eGFR) was calculated using the formula eGFR (mL/min/1.73 m^2^) = 186 × [serum creatinine (mg/dL)]^−1.154^  ×  [age (years)]^−0.203^ (× 0.742, if female) according to the modification of diet in renal disease (MDRD) equation [17]. Urinary albumin excretion was calculated using the formula UAE = albumin (mg)/creatinine (g) [1].

### 2.3. Statistical Analysis

All of the continuous data are presented as the mean values ± standard deviation (SD). The differences in the categorical variables across groups were analyzed by the chi-square test. The differences in the continuous variables across groups were analyzed by one-way analysis of variance (ANOVA). The change in each continuous variable within a group prior to and after this study was analyzed by paired *t* tests. The correlations between the changes in BDNF and those in the eGFR were assessed by Spearman's correlation. Multivariate linear regression analysis was used to analyze the factors associated with altered eGFR. Due to their skewed distributions, the triglyceride, UAE, and BDNF levels were logarithmically transformed (log) in our analyses. The statistical analyses were performed using SPSS 12.0 (SPSS, Inc., Chicago, IL, USA).

## 3. Results

**Figure 1:**
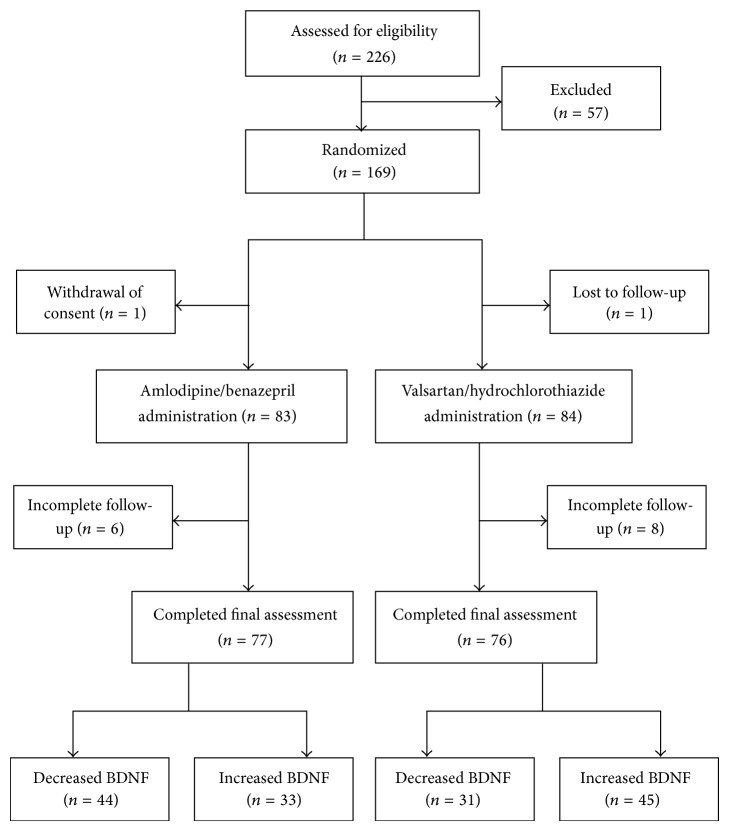
Flow diagram of the subjects included in the analyses.

Following a two-week discontinuance of antihypertensive drugs, 169 type 2 diabetic patients with hypertension were enrolled in twelve-week drug treatment study and randomized into one of two treatment groups. Of these 169 patients, 16 had incomplete follow-up and were excluded from further study. Our analyses included only 153 subjects with complete BDNF data prior to and after the study period, and 77 and 76 of these subjects were assigned to the amlodipine/benazepril and valsartan/hydrochlorothiazide groups, respectively (Figure 1). The systolic blood pressure was significantly reduced in both groups (from 141 ± 13 to 127 ± 15 mmHg, *P* < 0.001 in the amlodipine/benazepril group; from 140 ± 13 to 123 ± 13 mmHg, *P* < 0.001 in the valsartan/hydrochlorothiazide group, resp.). However, the reductions in the systolic pressure were not significantly different between these two study groups (*P* = 0.113). The diastolic blood pressure was also significantly reduced in both groups (from 86 ± 8 to 78 ± 8 mmHg, *P* < 0.001 in the amlodipine/benazepril group; from 87 ± 8 to 79 ± 10 mmHg, *P* < 0.001 in the valsartan/hydrochlorothiazide group, resp.). The reductions in the diastolic pressure were not significantly different between these two study groups (*P* = 0.563).

**Figure 2:**
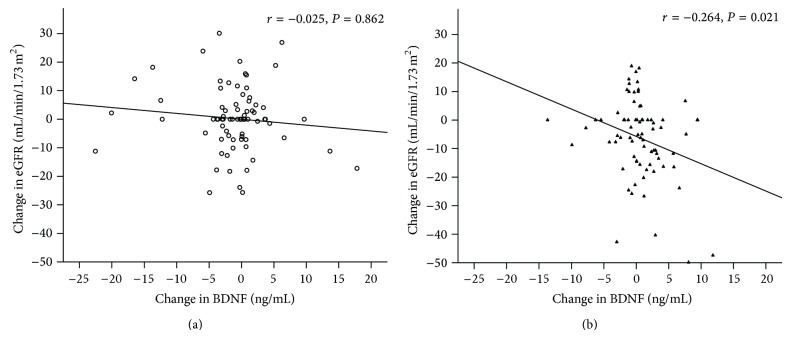
The correlations between alterations in the serum BDNF levels and the eGFR in subjects treated with (a) amlodipine/benazepril and (b) valsartan/hydrochlorothiazide.

There were no significant changes in the serum BDNF concentrations in either group (from 7.3 ± 6.7 to 6.2 ± 4.6 ng/mL, *P* = 0.209 in the amlodipine/benazepril group; from 5.2 ± 4.4 to 5.8 ± 4.7 ng/mL, *P* = 0.074 in the valsartan/hydrochlorothiazide group, resp.). There was no significant change in the eGFR in the amlodipine/benazepril group (80 ± 25 to 81 ± 25 mL/min/1.73 m^2^, *P* = 0.866). The eGFR was more significantly reduced in the valsartan/hydrochlorothiazide group (from 87 ± 26 to 81 ± 25 mL/min/1.73 m^2^, *P* < 0.001) than in the amlodipine/benazepril group (*P* = 0.002). The altered serum BNDF concentrations exhibited a significant inverse correlation with the eGFR in the valsartan/hydrochlorothiazide group (*r* = −0.264, *P* = 0.021) but not in the amlodipine/benazepril group (*r* = −0.025, *P* = 0.862) (Figure 2).

After the study, 44 subjects in the amlodipine/benazepril group exhibited decreased BDNF levels (−4.1 ± 5.3 ng/mL), whereas 33 subjects exhibited increased BDNF levels (3.3 ± 4.6 ng/mL). In the valsartan/hydrochlorothiazide group, 31 subjects exhibited decreased serum BDNF levels (−2.6 ± 3.1 ng/mL), whereas 45 subjects exhibited increased BDNF levels (3.0 ± 3.0 ng/mL) (Figure 1). The clinical characteristics of the subjects in these four groups are shown in Table 1. HbA1c was significantly increased in the subjects of the valsartan/hydrochlorothiazide group with increased BDNF compared with the subjects of the amlodipine/benazepril group with decreased BDNF or the patients with increased BDNF (*P* = 0.002 and 0.004, resp.). The triglyceride levels were also significantly higher in the subjects of the valsartan/hydrochlorothiazide group with increased BDNF compared to the subjects of the amlodipine/benazepril group with decreased BDNF or the subjects with increased BDNF (*P* < 0.001 and 0.049, resp.).

**Figure 3:**
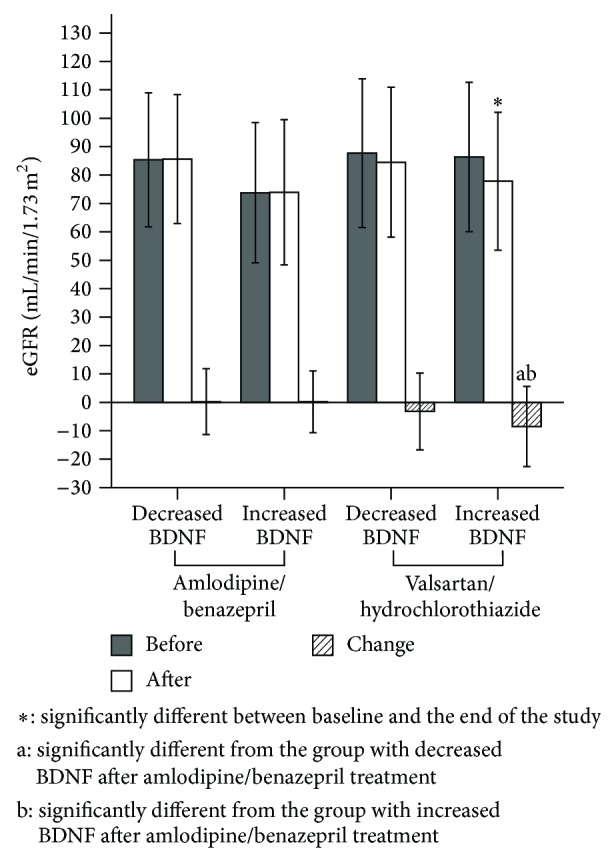
The eGFRs in the subjects grouped according to study drug treatment and change in BDNF prior to and after the study.

Figure 3 illustrates that the baseline eGFRs were not significantly different among these four groups (85.4 ± 23.6 mL/min/1.73 m^2^ in the amlodipine/benazepril group with decreased BDNF; 74.1 ± 24.4 mL/min/1.73 m^2^ in the amlodipine/benazepril group with increased BDNF; 87.7 ± 26.2 mL/min/1.73 m^2^ in the valsartan/hydrochlorothiazide group with decreased BDNF; and 88.0 ± 27.1 mL/min/1.73 m^2^ in the valsartan/hydrochlorothiazide group with increased BDNF; *P* = 0.071). After the study period, the changes in eGFR were statistically significant in the subjects of the valsartan/hydrochlorothiazide group with increased BDNF (−8.8 ± 14.9 mL/min/1.73 m^2^; *P* < 0.001) but not in the subjects of the valsartan/hydrochlorothiazide group with decreased BDNF (−3.2 ± 13.5 mL/min/1.73 m^2^, *P* = 0.198), the amlodipine/benazepril group with decreased BDNF (0.2 ± 11.6 mL/min/1.73 m^2^, *P* = 0.891), or the amlodipine/benazepril group with increased BDNF (0.5 ± 10.9 mL/min/1.73 m^2^, *P* = 0.923). Our multivariate regression analyses indicate that valsartan/hydrochlorothiazide treatment and the change in BDNF levels represent independent risk factors for reduced eGFR (Table 2).

## 4. Discussion

In this subgroup analysis of the ADDM study, a significant reduction in eGFR was observed in the subjects with increased serum BDNF who were treated with valsartan/hydrochlorothiazide but not in those with increased serum BDNF who were treated with amlodipine/benazepril. Because BDNF is associated with vascular contraction caused by sympathetic activation, the lower eGFRs observed might be attributed to reduced renal blood flow in the presence of increased BDNF [15, 18]. Diuretics with the potential to lower blood volume can exacerbate reduced blood flow in the glomerulus. Sympathetic activation induces calcium sensitivity in afferent arterioles and strengthens responsiveness to angiotensin II [19]. Conversely, dihydropyridine calcium channel blocker has an effect of sympathetic inhibition [20], which may induce dilation of afferent arterioles, and prevent renal damage caused by inadequate blood perfusion [20–24]. Therefore, eGFR was not significantly different between subjects who were treated with amlodipine/benazepril, regardless of the altered serum BDNF levels.

Alterations in renal nerves have been observed in diabetic subjects, and this neural damage might result in dysfunctional regulation of renal blood flow [25, 26]. Sympathetic overactivation has been reported to be associated with diabetic nephropathy [26]. BDNF can potentially increase sympathetic activation and energy expenditure in diabetic mice [27, 28]. It has also been reported that circulating BDNF was associated with energy status in diabetic subjects [29]. Furthermore, BDNF gene polymorphism is associated with vasoconstriction [30]. The BDNF-associated gene is also functionally responsible for type 2 diabetic nephropathy, as determined through previously reported microarray analyses [31]. However, in this subgroup analysis of the ADDM study, we observed an inverse correlation between changes in eGFR and changes in the serum BDNF. The causal effect could not be clarified because of the initial study design of the clinical trial for antihypertensive drugs. Further investigation of the mechanisms underlying BDNF-associated diabetic nephropathy is needed.

In addition to maintaining the eGFR, amlodipine/benazepril also significantly reduced the HbA1c and serum triglyceride levels and increased HDL cholesterol compared with valsartan/hydrochlorothiazide, as previously reported in the ADDM study [9]. However, the alterations in HbA1c, triglycerides, and HDL cholesterol were not independent factors for a decline in eGFR in our analysis. Furthermore, BDNF has been reported to play a role in energy homeostasis and might be associated with insulin sensitivity [30, 32]. HbA1c has been shown to decrease after the injection of BDNF [27]. The circulating BDNF concentrations are also decreased in subjects with obesity or metabolic syndromes [29, 33]. In our analysis, there were no significant differences in body weight, HbA1c, triglyceride, and HDL cholesterol between the subjects after valsartan/hydrochlorothiazide treatment, regardless of the serum BDNF level alterations. Similar findings were observed in the subjects who were treated with amlodipine/benazepril. The improvement in the glucose and lipid profiles might not have directly resulted from the altered BDNF levels observed during this short-term study.

There were some limitations in this study. First, only subjects with completed BDNF assessments were enrolled in the analysis. Therefore, the data from 14 subjects who had incomplete follow-ups after taking the study drugs (in the intention totreat population) were not included. However, the subjects without complete follow-up did not significantly differ in age, gender, blood pressures, HbA1c, triglycerides, cholesterol, BDNF, or eGFR from those who were included (all *P* values > 0.05). Second, apart from the differences in calcium channel blockers and diuretics, ACE inhibitors and ARBs might elicit different effects on diabetic nephropathy [34]. Third, the serum concentrations and not the local expression levels of BDNF were detected in this study. We could not exclude other systemic factors that might confound the eGFR. Fourth, neither sympathetic activity nor renal blood flow was directly assessed in the study. It remains unclear whether renal sympathetic denervation can prevent chronic kidney disease in subjects with hypertension [35, 36]. Furthermore, due to the short study duration, end-stage renal disease events were not assessed in this analysis. The effects of BDNF on chronic kidney disease require a long-term follow-up study.

In conclusion, increased serum BDNF levels are associated with reduced eGFR in type 2 diabetic subjects treated with antihypertensive drugs. However, the potential effects of BDNF are not observed in subjects treated with a combination of an ACE inhibitor and a calcium channel blocker.

## Figures and Tables

**Table 1:** Characteristics of the subjects grouped by drug treatment and changes in the serum BDNF levels prior to and after the study.

	Amlodipine/benazepril		Valsartan/hydrochlorothiazide		
	Decreased BDNF	Increased BDNF	Decreased BDNF	Increased BDNF	*P* ^#^
	(*N* = 44)	(*N* = 33)	(*N* = 31)	(*N* = 45)	
Age (years)	60 ± 10	61 ± 11	60 ± 10	57 ± 11	0.329
Gender (male)	26 (59.1%)	16 (48.5%)	20 (64.5%)	30 (66.7%)	0.401
Body weight (kg)					
Baseline	71.2 ± 14.1	65.8 ± 13.7	68.8 ± 11.5	72.1 ± 14.0	0.173
End of trial	70.8 ± 13.6	65.6 ± 13.3	68.2 ± 11.3	72.0 ± 14.0	
Change from baseline	−0.4 ± 1.7	−0.2 ± 1.3	−0.6 ± 2.0	−0.2 ± 1.4	0.653
BMI (kg/m^2^)					
Baseline	27.2 ± 4.2	25.5 ± 3.1	25.8 ± 3.4	27.2 ± 4.4	0.114
End of trial	27.1 ± 4.0	25.5 ± 3.0	25.6 ± 3.6	27.1 ± 4.4	
Change from baseline	−0.1 ± 0.7	−0.1 ± 0.5	−0.2 ± 0.7	−0.1 ± 0.5	0.796
Waist circumference (cm)					
Baseline	92.1 ± 10.4	88.6 ± 9.9	87.7 ± 9.2	92.3 ± 11.4	0.120
End of trial	91.7 ± 10.0	89.2 ± 9.8^*^	88.0 ± 9.3	92.7 ± 11.7	
Change from baseline	−0.4 ± 2.4	0.6 ± 1.8	0.4 ± 2.5	0.3 ± 2.8	0.243
Systolic BP (mmHg)					
Baseline	141 ± 13	142 ± 14	138 ± 10	141 ± 14	0.504
End of trial	126 ± 13^***^	130 ± 16^***^	118 ± 13^***^	126 ± 13^***^	
Change from baseline	−16 ± 11	−13 ± 14	−20 ± 11	−15 ± 12	0.104
Diastolic BP (mmHg)					
Baseline	86 ± 8	86 ± 8	86 ± 7	89 ± 9	0.298
End of trial	78 ± 9^***^	80 ± 6^***^	77 ± 10^***^	81 ± 10^***^	
Change from baseline	−8 ± 7	−6 ± 7	−8 ± 9	−8 ± 10	0.633
Fasting glucose (mmol/L)					
Baseline	8.4 ± 2.3	8.6 ± 2.4	8.6 ± 2.2	9.1 ± 2.1	0.484
End of trial	8.3 ± 2.2	8.0 ± 2.2	8.9 ± 2.8	8.8 ± 2.2	
Change from baseline	−0.1 ± 2.4	−0.6 ± 3.0	0.4 ± 2.7	−0.3 ± 2.3	0.502
HbA1c (%)					
Baseline	8.2 ± 1.0	7.9 ± 1.0	7.7 ± 0.8	8.0 ± 0.9	0.171
End of trial	7.9 ± 1.1	7.9 ± 1.3	7.9 ± 1.2	8.4 ± 1.3^*^	
Change from baseline	−0.3 ± 0.7	0.0 ± 0.8	0.2 ± 0.6^a^	0.4 ± 1.1^ab^	0.002
Triglyceride (mmol/L)					
Baseline	1.7 ± 0.9	1.6 ± 0.7	2.2 ± 1.6	2.1 ± 2.0	0.367
End of trial	1.6 ± 0.9	1.5 ± 0.6	2.3 ± 1.6	2.6 ± 2.2^*^	
Change from baseline	−0.1 ± 0.7	−0.1 ± 0.5	0.1 ± 0.8	0.5 ± 1.2^ab^	0.010
Total cholesterol (mmol/L)					
Baseline	4.7 ± 0.7	5.0 ± 1.0	5.0 ± 0.9	4.7 ± 0.8	0.209
End of trial	4.7 ± 0.8	5.0 ± 1.0	4.7 ± 0.7	4.9 ± 0.9^*^	
Change from baseline	0.1 ± 0.7	0.0 ± 0.7	−0.3 ± 0.9^b^	0.2 ± 0.6^c^	0.041
LDL cholesterol (mmol/L)					
Baseline	2.8 ± 0.5	3.1 ± 0.8	2.9 ± 0.8	2.7 ± 0.7	0.143
End of trial	2.9 ± 0.6	3.1 ± 0.7	2.8 ± 0.7	2.8 ± 0.8	
Change from baseline	0.1 ± 0.6	0.0 ± 0.6	−0.1 ± 0.5	0.0 ± 0.6	0.632
HDL cholesterol (mmol/L)					
Baseline	1.0 ± 0.2	1.1 ± 0.3	1.1 ± 0.3	1.1 ± 0.3	0.908
End of trial	1.1 ± 0.3^**^	1.1 ± 0.3	1.0 ± 0.3	1.1 ± 0.3	
Change from baseline	0.1 ± 0.2	0.0 ± 0.2	−0.1 ± 0.2^a^	0.0 ± 0.2	0.016
Serum creatinine (*µ*mol/L)					
Baseline	81.4 ± 20.7	91.9 ± 26.8	82.3 ± 25.5	83.8 ± 29.5	0.307
End of trial	80.6 ± 19.7	91.4 ± 26.7	86.7 ± 31.5^*^	92.8 ± 35.1^*^	
Change from baseline	−0.8 ± 9.3	−0.4 ± 10.1	4.4 ± 11.4	9.0 ± 17.3^ab^	0.001
eGFR (mL/min/1.73 m^2^)					
Baseline	85.4 ± 23.6	74.1 ± 24.4	87.7 ± 26.2	88.0 ± 27.1	0.071
End of trial	85.6 ± 22.7	74.6 ± 25.4	84.5 ± 26.4	79.2 ± 26.0^***^	
Change from baseline	0.2 ± 11.6	0.5 ± 10.9	−3.2 ± 13.5	−8.8 ± 14.9^ab^	0.003
Hemoglobin (g/L)					
Baseline	13.6 ± 1.7	13.3 ± 2.1	13.9 ± 2.2	13.6 ± 1.8	0.586
End of trial	13.4 ± 1.5^*^	12.8 ± 1.9^**^	13.7 ± 1.9	13.4 ± 1.8	
Change from baseline	−0.3 ± 0.7	−0.4 ± 0.8	−0.2 ± 0.9	−0.2 ± 1.0	0.587
Platelets (10^9^/L)					
Baseline	243.4 ± 61.1	250.6 ± 72.2	256.7 ± 86.0	249.7 ± 66.4	0.882
End of trial	249.0 ± 59.7	262.7 ± 71.9	253.2 ± 62.8	261.6 ± 69.0^*^	
Change from baseline	5.5 ± 25.4	12.1 ± 37.9	−3.5 ± 50.2	11.9 ± 37.4	0.282
UAE (mg/g)					
Baseline	162 ± 208	188 ± 255	331 ± 85	332 ± 666	0.848
End of trial	214 ± 315	162 ± 235	194 ± 555^*^	262 ± 605^*^	
Change from baseline	52 ± 282	−25 ± 135	−137 ± 358^a^	−70 ± 343	0.047
BDNF (ng/mL)					
Baseline	9.3 ± 7.9	4.6 ± 3.1^a^	6.4 ± 4.6	4.3 ± 4.0^ac^	<0.001
End of trial	5.2 ± 4.2^***^	7.9 ± 5.4^***^	3.9 ± 2.9^***^	7.3 ± 5.4^***^	
Change from baseline	−4.1 ± 5.3	3.3 ± 4.6^a^	−2.6 ± 3.1^b^	3.0 ± 3.0^ac^	<0.001

BDNF, brain-derived neurotrophic factor; BMI, body mass index; BP, blood pressure; eGFR, estimated glomerular filtration rate; HbA1c, glycated hemoglobin; HDL, high-density lipoprotein; LDL, low-density lipoprotein; UAE, urinary albumin excretion.

Due to their skewed distributions, BDNF, triglyceride, and UAE were logarithmically transformed (log) in the analyses.

^#^ *P* values among the four groups.

^*^ *P* < 0.05, ^**^ *P* < 0.01, and ^***^ *P* < 0.001 compared to the baseline.

^a^significantly different from the group with decreased BDNF after amlodipine/benazepril treatment.

^b^significantly different from the group with increased BDNF after amlodipine/benazepril treatment.

^c^significantly different from the group with decreased BDNF after valsartan/hydrochlorothiazide treatment.

**Table 2:** Multivariate regression analysis showing an independent association between altered serum BDNF levels and altered eGFR after the study^∗^.

	*β* ^1^	*B* ^2^	95% CI	*P*
Valsartan/hydrochlorothiazide	−0.204	−5.328	(−9.633, −1.022)	0.016
ΔBDNF (ng/mL)	−0.189	−0.481	(−0.887, −0.076)	0.020
ΔBMI (kg/m^2^)	0.140	2.970	(−0.287, 6.228)	0.074
Δtriglyceride (mmol/L)	0.138	1.998	(−0.375, 4.371)	0.098
ΔHbA1c (%)	−0.084	−1.281	(−3.720, 1.157)	0.301
Δplatelets (10^5^/*μ*L)	−0.059	−2.043	(−7.469, 3.383)	0.458
Δtotal cholesterol (mmol/L)	−0.051	−0.925	(−3.781, 1.931)	0.523
Δsystolic BP (10 mmHg)	−0.018	−0.196	(−1.841, 1.450)	0.814

^∗^after adjusting for the age, gender, and baseline eGFR.

^1^ *β*: standardized coefficient, ^2^ *B*: linear regression coefficient.

Δ: variable after treatment, variable before treatment

BDNF, brain-derived neurotrophic factor; BMI, body mass index; BP, blood pressure, eGFR, estimated glomerular filtration rate; HbA1c, glycated hemoglobin.

## Abbreviations

ACE: Angiotensin-converting enzyme
ADDM study: Amtrel and co-Diovan in type 2 diabetes mellitus hypertension patients with microalbuminuria study
ANOVA: One-way analysis of variance
ARB: Angiotensin II receptor blocker
BDNF: Brain-derived neurotrophic factor
BMI: Body mass index
BP: Blood pressure
eGFR: Estimated glomerular filtration rate
HbA1c: Glycated hemoglobin
HDL: High-density lipoprotein
LDL: Low-density lipoprotein
log: Logarithmically transformed
MDRD: Modification of diet in renal disease
SD: Standard deviation
UAE: Urinary albumin excretion.
